# The range of the mange: Spatiotemporal patterns of sarcoptic mange in red foxes (*Vulpes vulpes*) as revealed by camera trapping

**DOI:** 10.1371/journal.pone.0176200

**Published:** 2017-04-19

**Authors:** David Carricondo-Sanchez, Morten Odden, John D. C. Linnell, John Odden

**Affiliations:** 1 Faculty of Applied Ecology and Agricultural Sciences, Hedmark University of Applied Sciences, Koppang, Norway; 2 Norwegian Institute for Nature Research, Trondheim, Norway; Universidade de Aveiro, PORTUGAL

## Abstract

Sarcoptic mange is a widely distributed disease that affects numerous mammalian species. We used camera traps to investigate the apparent prevalence and spatiotemporal dynamics of sarcoptic mange in a red fox population in southeastern Norway. We monitored red foxes for five years using 305 camera traps distributed across an 18000 km^2^ area. A total of 6581 fox events were examined to visually identify mange compatible lesions. We investigated factors associated with the occurrence of mange by using logistic models within a Bayesian framework, whereas the spatiotemporal dynamics of the disease were analysed with space-time scan statistics. The apparent prevalence of the disease fluctuated over the study period with a mean of 3.15% and credible interval [1.25, 6.37], and our best logistic model explaining the presence of red foxes with mange-compatible lesions included time since the beginning of the study and the interaction between distance to settlement and season as explanatory variables. The scan analyses detected several potential clusters of the disease that varied in persistence and size, and the locations in the cluster with the highest probability were closer to human settlements than the other survey locations. Our results indicate that red foxes in an advanced stage of the disease are most likely found closer to human settlements during periods of low wild prey availability (winter). We discuss different potential causes. Furthermore, the disease appears to follow a pattern of small localized outbreaks rather than sporadic isolated events.

## Introduction

Sarcoptic mange is a parasitic disease caused by the burrowing mite *Sarcoptes scabiei* (L., 1758, Latrielle, 1802). This disease is widely distributed around the world and it affects a wide range of mammalian species. Sarcoptic mange has been reported in Eurasian lynx (*Lynx lynx*) [1], European rabbits [2], Iberian ibex (*Capra pyrenaica*) [3] and different wolf populations (*Canis lupus*) [4–6] in Europe. It has also been reported in coyotes (*Canis latrans*) in North America [7], wombats (*Vombatus ursinus*) in Australia [8, 9] or recently in Kenyan giraffes (*Giraffa camelopardalis reticulata*) [10]. Overall, it affects over 100 mammalian species and the red fox (*Vulpes vulpes*) is one of the most important hosts [11].

Due to the burrowing activity of the mite, the host can develop thickening of the skin (hyperkeratosis), irritation of the skin (erythema), dermatitis (seborrhea) and patchy hair loss (alopecia) as the most common symptoms [12–15]. In species like the red fox, death usually occurs within 2–4 months [16, 17].

Epizootics of sarcoptic mange can affect the dynamics and behaviour of the affected population, for example by increasing natural mortality rates [18] or altering the territoriality of the animals [19]. These epizootics can be especially dramatic in endangered and fragmented populations [20, 21], and can even lead to extinction [22]. Host populations can eventually recover from epizootics of mange, although times are relatively long, ranging from 15 to 20 years [7, 23, 24]. After recover, the disease can enter an enzootic phase in which the parasite is still, locally or globally, present in the population [14, 25]. At this stage, the disease can re-emerge in short-term fluctuations or even causing new outbreaks [24, 26].

Sarcoptic mange has received much attention in studies of red foxes [13, 14, 23, 27], mainly due to dramatic outbreaks like the one that occurred in Scandinavia in the late 1970´s, or the later and more localized outbreak in Bristol (UK) in 1994 [28]. The social behaviour of this species [29] and their natal-dispersal and exploratory movements facilitate these outbreaks [30–32]. These behaviours promote the spread of the disease either by direct physical contact or indirectly (e.g. from sharing dens) [33].

In Fennoscandia, the disease spread from southeastern Finland in 1967 to Sweden where the first mangy foxes were reported in 1972, and in Norway in 1975 [16, 25]. The outbreak reduced red fox numbers drastically [23, 34]. The reduction in the red fox population revealed a pronounced impact on grouse populations [35], and on other alternative prey species such as the mountain hare (*Lepus timidus*) and roe deer (*Capreolus capreolus*) fawns [23, 36].

Monitoring enzootic diseases within a population is especially important in social species like the red fox [29] that utilize resources of anthropogenic origin as a supplement to their diet [37, 38]. An advanced state of sarcoptic mange undermines the body condition of red foxes [17, 26, 27], which in turn, can lead to a decrease in their hunting success or a poor ability to compete for carcasses [27, 39]. These individuals may be forced to look for “easier” food resources such as garbage. Mangy foxes close to human settlements might represent a threat to the health of wild and companion animals or livestock, and even to humans [40–43]. It is therefore important to understand the spatiotemporal dynamics of the disease in order to assess this threat and to take the necessary measures.

We used the non-invasive technique of camera trapping in order to investigate the spatiotemporal dynamics of sarcoptic mange in red foxes. Traditionally, researchers have inspected hunting bags or used physical capture-recapture techniques to study this disease on wildlife. This may lead to biases since red foxes with poor body condition might show a different trapability as well as being easier targets for hunters. To date, very few studies have used camera traps to detect or monitor sarcoptic mange outbreaks. Nevertheless, some studies have applied this tool along with laboratory techniques to assess sarcoptic mange in wolves [4, 5]. Borchard et al. [9] also used motion-sensing cameras to study the activity of mangy wombats. However, in our study we carried out the most extensive camera trap assessment of sarcoptic mange in wildlife that have been conducted so far.

In our study, we used five years of camera trap data to investigate large-scale patterns of sarcoptic mange apparent prevalence in red foxes. We hypothesize that patterns of spatial distribution differ between healthy and infected red foxes, as the latter may be more dependent on easily accessible food resources due to the negative impact of sarcoptic mange on body condition [17, 26, 27]. Therefore, we predicted that red foxes showing evident symptoms of sarcoptic mange would occur in areas closer to human settlements more frequently than healthy red foxes. Our second hypothesis states that if the disease is in an enzootic state in Norway, and given the social character of the red fox there are frequent intraspecific interactions that facilitate transmission, then the dynamic of the disease should follow patterns of local outbreaks rather than random isolated events. This is the case in other fox populations where sarcoptic mange is in an enzootic state [24, 26].

## Material and methods

### Study area

**Fig 1:**
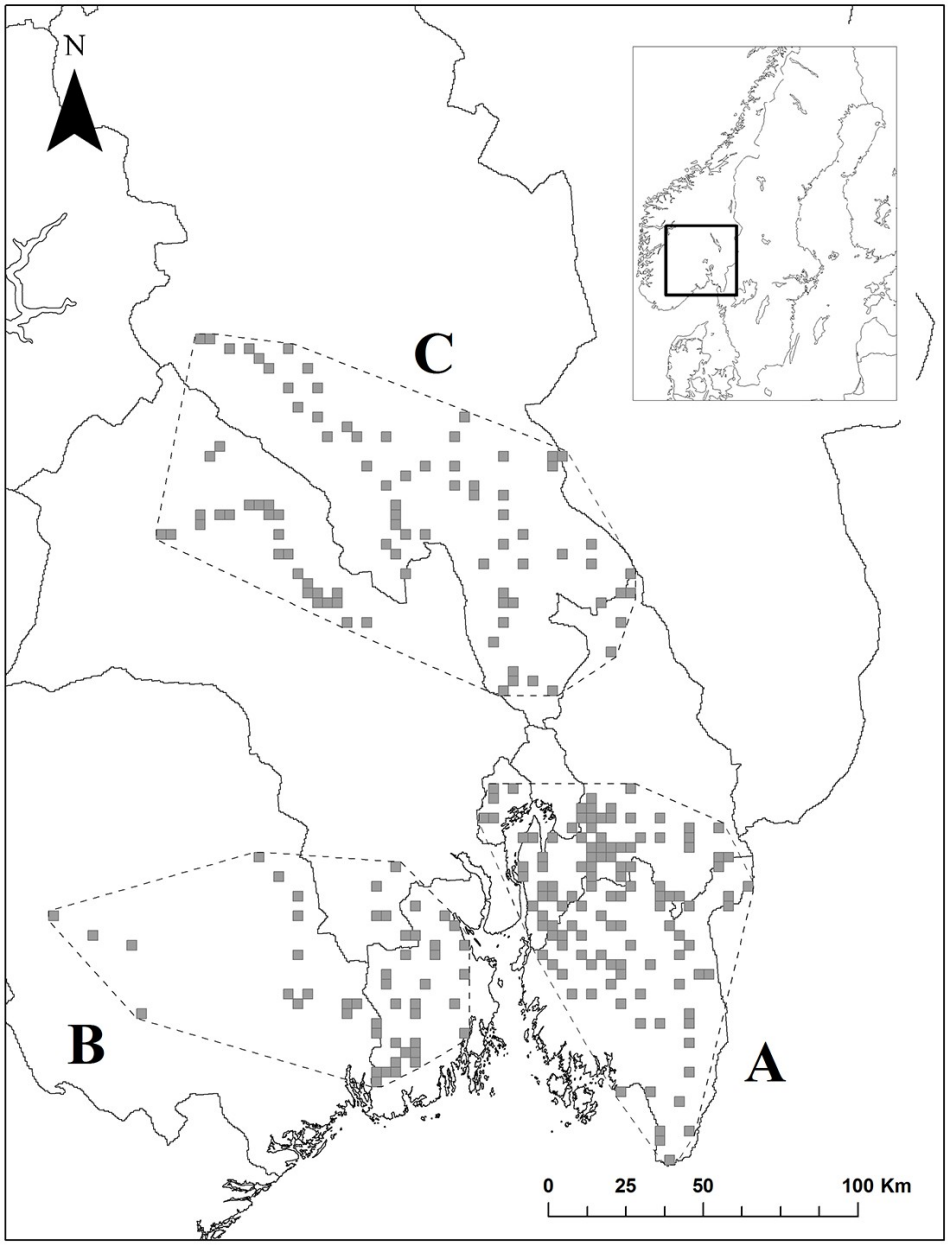
Study area. Location and extent of three areas (A, B, and C) in southeastern Norway where we studied spatiotemporal patterns of sarcoptic mange in red foxes with camera traps. Gray squares are sampled 10 km^2^ cells. Dashed lines are minimum convex polygons around camera locations in each of the study areas.

We used data from a long-term camera trapping study of Eurasian lynx (Scandlynx, http://scandlynx.nina.no). The study was conducted in the southeastern part of Norway (Fig 1), in three study areas covering a total of ca. 18000 km^2^. Area A covered ca. 6573 km^2^ to the east of Oslo fjord, and included parts of the counties of Oslo, Akershus and Østfold. Area B was situated west of Oslo fjord and covered ca. 7145 km^2^. It included parts of the counties of Vestfold, Buskerud and Telemark. Area C covered ca. 4474 km^2^, and was situated north of the other two areas and included parts of the counties of Oppland and Buskerud (Fig 1). For cameras placed in national park land, permissions were obtained from the Country administrations in Oslo and Akershus and Østfold. Most cameras were placed on private land, and here permissions were obtained from the landowner. No other specific permission are required for these locations according to Norwegian law.

The study areas cover a gradient from periurban or suburban areas to a fragmented forest—farmland mosaic in the areas closest to Oslo and Oslo fjord, to forest dominated landscapes towards the north, west, and east [44]. The forests are mainly composed of Norwegian spruce (*Picea abies*), Scots pine (*Pinus silvestris*), interspersed with deciduous species such as hoary alder (*Alnus incana*) and birch (*Betula pubescens*) [44].

### Camera trapping

The camera trapping was initiated to complement the lynx population monitoring program in areas where the commonly used method of snowtracking was inefficient due the unpredictable snow conditions [45, 46]. The project started as a pilot study in Area A during the winter of 2010/2011, and in Area B during the summer of 2011. Later, the project was fully implemented in Area A in the winter of 2011/2012, in the summer of 2012 in Area B, and in the winter of 2013/2014 in area C. Areas A and B were expanded in the summer of 2014 and Area A expanded again in the winter of 2015. We used data from the winter of 2010/2011 to the summer of 2015.

Cameras were dispersed across a grid overlaid over the study areas to ensure that all parts of the areas were covered. However, within each grid cell the specific locations were selected to maximise the probability of detecting lynx; typically forest roads, human and game trails, or natural movement routes at the base of steep cliffs. At each location, a camera trap was placed 30–60 cm above the ground pointing 90 degrees towards the path. All sites had one Reconyx camera with infrared flash (Models HC500, HC600, PC800, and PC900). In Area A and B most locations had an additional camera with white flash (Cuddeback Capture or Reconyx PC850) on the other side of the path so that pictures of both sides of the lynx could be photographed for individual recognition. Previous studies have shown that the use of lures do not affect population or individual parameters like immigration rates, maximum movement distance or temporal activity [47, 48]. On the other hand, not using attractants may underestimate the number of individuals [49]. Therefore, we added some drops of lure (catnip oil, valerian oil or beaver castorium) in front of the camera. The cameras ran for 24 hours a day, all year round, and were set up to be triggered by movement and take three pictures per event. The camera traps were checked once a month, to download pictures, change batteries and add lure. All the pictures from these camera traps were uploaded online at http://viltkamera.nina.no.

**Fig 2:**
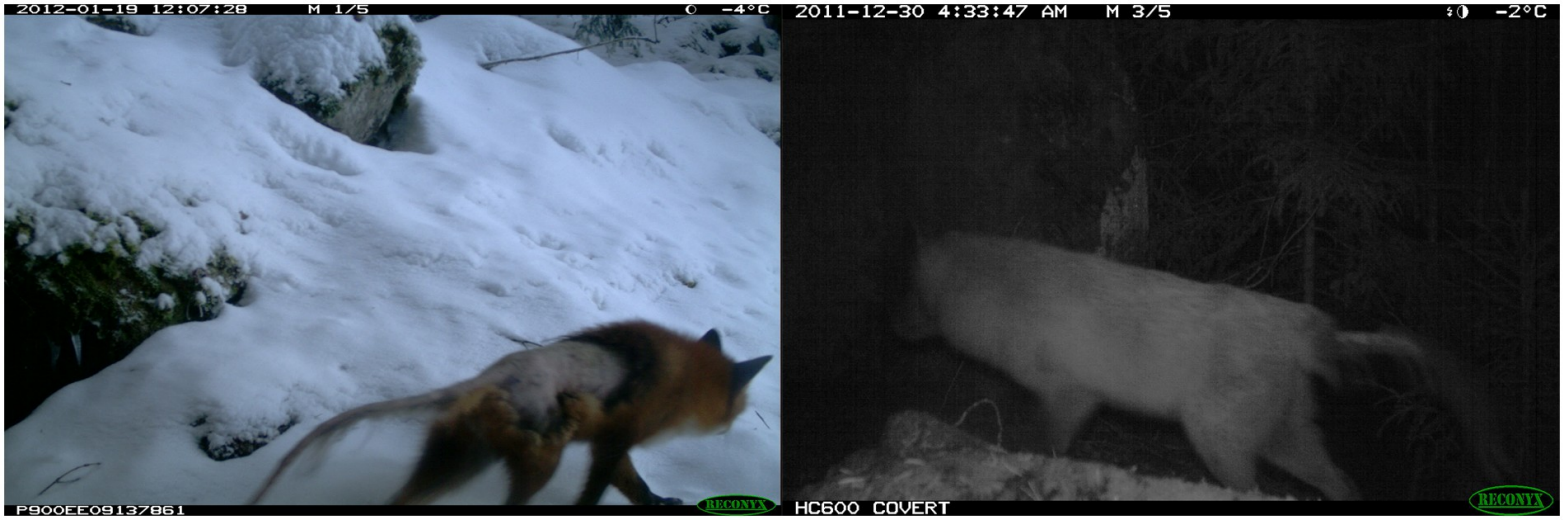
An example of two camera trapped red foxes with mange-compatible lesions.

All the pictures obtained from the cameras were visually checked online and from the raw data obtained from Scandlynx. Each group of three pictures taken consecutively by a camera was considered a single event. Red fox events were selected and visually checked for mange-compatible lesions like alopecia in the lower back and tail of the animal as previously described by Oleaga et al. [5] (e.g. Fig 2). A single researcher conducted a first round of identification, and subsequently, two other researchers rechecked events that had been difficult to classify. Red foxes with low or moderate infections can be more difficult to identify in locations with only infrared cameras than in locations with both infrared and white flash cameras. We classified as infected only red foxes with distinct patterns of mange-compatible lesions. For example, we were careful not to classify as infected individuals that only had poor body condition, but no alopecic patches. Likewise, we did not consider individuals showing only small alopecic patches as infected, as this could be other external wounds. Furthermore, we disregarded pictures that were out of focus or burned, and hence, we avoided potential bias caused by the use of cameras with different types of flash.

### Analyses

We used data from 305 camera trap locations working for 146607 effective trapping nights from winter 2010/2011 to summer 2015. We used all the cameras with red fox detection events for the analyses.

Pseudoreplication is a common problem when working with photographic material from naturally unmarked species. For this study, we created a fishnet grid of square cells of 10 km^2^ in order to group cameras too close together. This cell size exceeded the average area of red fox home ranges (7.06 km^2^; [50]). Following this method, we ensured spatial independence of observations from each grid cell. Red foxes usually die, from two to four months after infection; although this time can vary [16, 17, 27]. Accordingly, in order to investigate how the disease developed over time and to ensure temporal independence among observations, we divided the total study period into six-months time periods. Winter periods spanned from October until March (roughly the months with snow cover) and summer periods from April to September (snow free months).

#### Apparent prevalence

We defined prevalence as the proportion of camera trapping events of red foxes with mange-compatible lesions over the total number of red fox events. However, since we did not conduct serological tests to confirm that the infected cases corresponded to actual sarcoptic mange cases, we considered the prevalence calculated in this study as apparent prevalence. We calculated the apparent prevalence of the disease for each of the three areas in each of the time periods and calculated the Bayesian credible intervals (CRI hereafter) by using Beta (0.5, 0.5) prior distributions of the probability of success (i.e. a mangy fox event). For this we used the function binom.bayes in the R package binom [51].

#### Model selection

In each 10 km^2^ cell, we calculated (i) the proportion of agricultural land, (ii) the proportion of the area covered by human settlements, and (iii) human population density (Table 1). For the two former variables, we extracted data from the Vegetation Map of Norway [52], and for the latter, we retrieved data from a 5x5 km resolution map of population density from Statistics Norway (http://kart.ssb.no). For each camera location, we calculated (iv) the distances to the nearest settlement. We used the software ArcGis 10.3 [53] for all GIS analyses. In addition to the covariates listed above, we included (v) the time each event occurred expressed as the number of months that had passed since the initiation of the study, (vi) the frequency of red fox photographic events, i.e. the average number of fox events per day in each six months time period and area (A, B or C), (vii) season (winter or summer) and (viii) area (A, B or C; Table 1).

**Table 1:** Variables used in logistic models of the occurrence of red foxes showing mange-compatible lesions in southeastern Norway.

Name of variable	Definition of variable	Type of variable
Dist_Settle	Distance to closest settlement from camera location in kilometres.	Numeric
Agriculture	Percentage of the grid cell covered by agriculture land.	Numeric
HumSettle	Percentage of the grid cell covered by human settlements.	Numeric
Hum_pop	Human population density with 5km resolution.	Numeric
Time	Continuous variable indicating the number of months since the beginning of the study (from 1 to 60).	Numeric
Avg_pic_day	Average number of events per area and per day in each time period.	Numeric
Season	Winter (October to March) or Summer (April-September).	Factor
Area	Factor designating the three study areas.	Factor
Grid_id	Individual id for the 10 km^2^ sampling grid cells.	Factor
Time period	Number of the six-months time period in which the study was divided	Numeric

We ran Pearson correlation tests for all combinations of covariates. The correlation coefficients (r) ranged from -0.26 to 0.31, and we considered these values too small to indicate dependency among the covariates. Therefore, we included all the variables in the models. We also produced spline correlograms in order to check for spatial autocorrelation. The spline correlogram indicated some spatial autocorrelation that we corrected by including individual cell id as random effect in the models. All the numeric variables were standardized by $({\text{x}}_{\text{i}}-\bar{\text{X}})/\left(2\times \text{SD}\right)$ [54].

We created a list of nine candidate models based on our predictions. We included models with different combinations of covariates related to activity and distribution of humans, red fox distribution, time and area (Table 2). We also constructed models that included interactions between season and the most relevant human related variables, and interactions between time and area. We included a *null model* of variation around the grand mean (Table 2). We used a grid cell id and time period as random factors to avoid spatial and temporal autocorrelation of the response respectively.

**Table 2:** Set of candidate logistic models for the occurrence of red foxes showing mange-compatible lesions in southeastern Norway.

	NULL	Model1	Model2	Model3	Model4	Model5	Model6	Model7	Model8
Agriculture			*	*			*		
Hum_Settle			*	*			*		
Hum_pop			*	*					
Season			*	*		*	*	*	*
Dist_Settle			*	*	*	*	*	*	*
Avg_Pict_day			*	*					
Time			*	*				*	*
Area			*	*				*	
Time:Area				*				*	
Dist_Settle:Season				*		*	*	*	*
Agriculture:Season				*			*		
Random:Grid_id		*	*	*	*	*	*	*	*
Random: Time period		*	*	*	*	*	*	*	*
LOO	1510.05	1185.26	1184.29	1184.29	1185.29	1181.20	1185.30	1179.95	1179.42

See Table 1 for the definitions of the variables. LOO are leave-one-out values from the regressions.

We used each event as an observation of a binomial process (presence of mange-compatible lesion on the fox or not) and modelled them in a Bayesian framework using the brm function in the brm package [55] in R (www.r-project.com), version 3.3.1. We used normal distributions with mean = zero and standard deviation = 10 as non-informative priors for the explanatory variables and a t-student distribution with three degrees of freedom as a non-informative prior for the random effects. Models were fitted using four chains with 3000 iterations each, of which 1000 were discarded as a burn-in process. Following this procedure, we obtained a total of 8000 posterior samples per model. We used leave-one-out-cross correlation values (LOO) as indicators of model fit and used it for model selection [56]. We visually checked for the convergence of the models by looking at the density distribution plots and by using the Gelman and Rubin´s converge diagnosis [57] represented by Ȓ in our analyses.

### Spatiotemporal scan analysis

For the detection of potential clusters of disease occurrence, we used the Kulldorff algorithm [58] based on a Bernouilli probability model. This algorithm is implemented in the scan analysis software SATSCAN (www.satscan.org), version 9.4.2, and analyses three dimension cylindrical windows centered at the discrete locations of the study. It takes into account the positive and negatives events in the scanned cylindrical window where the height of the cylinder represents the time and the base is the space. Both dimensions are flexible, meaning that the algorithm will analyse any area up to a specified maximum and every time block within the study period [59]. The scan window moves across the study area and the maximum likelihood ratio of the potential clusters are compared to the maximum likelihood of random simulations of the data under the null hypothesis obtained by Monte Carlo simulations [59]. We specified a maximum temporal and spatial cluster size of a 50% of the population, and a maximum cluster radius of 50 km. We used 999 Monte Carlo simulations to compute p-values at the 0.05 level.

Additionally, we investigated if resulted clustered locations were closer to human settlements than other locations. In order to do this, we fitted a linear regression within a Bayesian framework with distance to settlement as a response variable and the explanatory variable indicating whether a location belonged to the most likely cluster or not. We used the same model settings as in the previous model selection analyses.

## Results

We obtained red fox photographs from 224 of the 305 camera locations. In total, 6579 red fox events were identified, among which 173 showed mange-compatible lesions (S1 Appendix). The camera locations were located within 201 cells of 10 km^2^. We detected foxes with mange-compatible lesions at least once in 19.40% of these cells over the study period.

### Apparent prevalence

**Fig 3:**
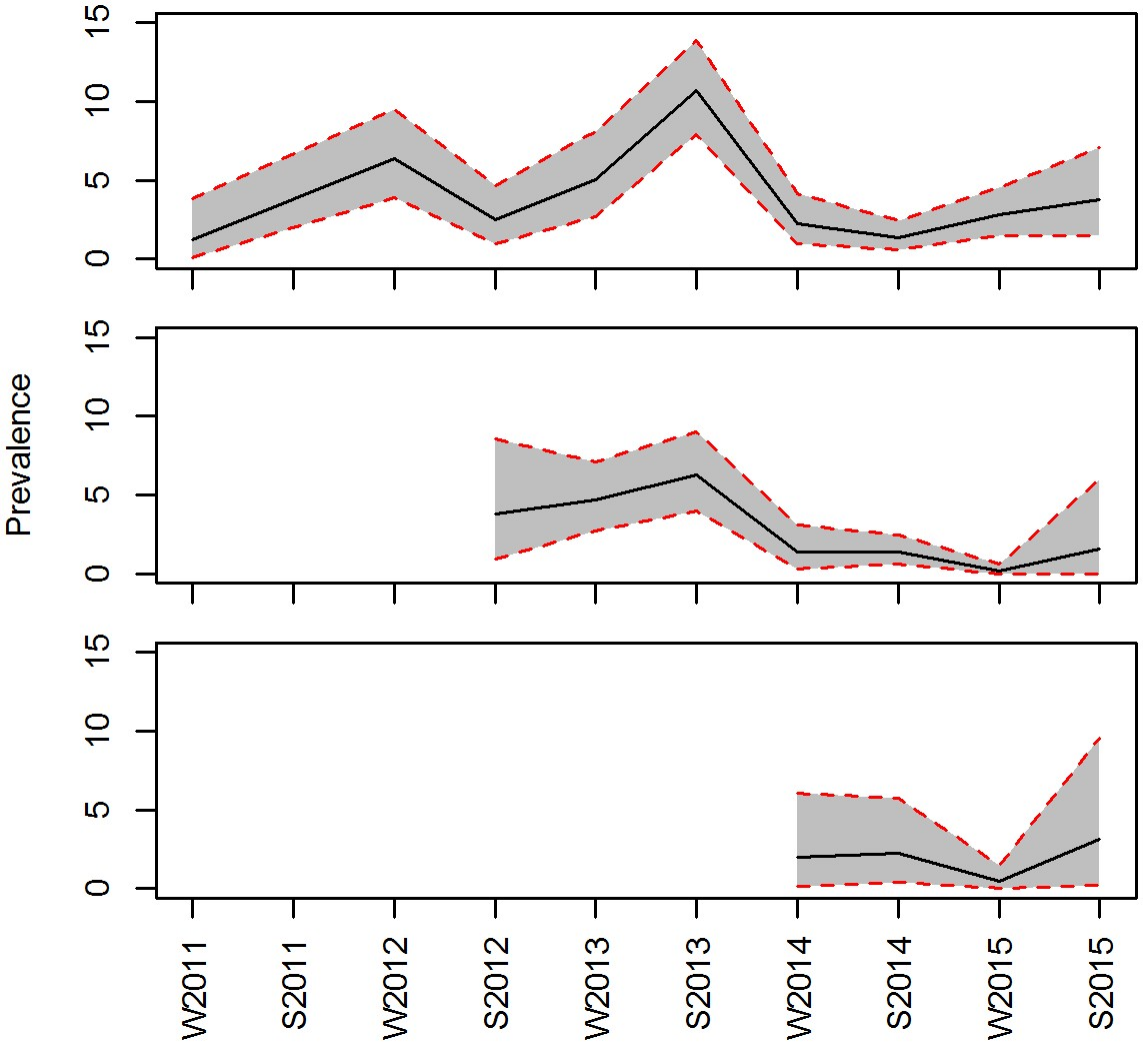
Apparent prevalence of sarcoptic mange in red foxes in southeastern Norway during the five years of study. Red dashed lines and grey area represent the 95% credible intervals.

The apparent prevalence of sarcoptic mange in the study areas fluctuated over the time of the study. The mean overall prevalence was 3.15% CRI [1.25, 6.37]. It reached a maximum of 10.70% CRI [7.9, 13.83] during the summer of 2013 in Area A that corresponded with a maximum occurrence in Area B at 6.28% CRI [4.01, 9.01]. The maximum in area C was 3.12% CRI [2.30, 9.51]. The minimum was 0.01% CRI [0, 0.06] in Area B in the winter of 2015 (Fig 3).

### Model selection

All models visually converged and presented a Ȓ of one. Models seven and eight had the lowest LOO value (Table 2), and the two models differed only in that the former included the covariate area and the interaction between time and area. Therefore, we selected model eight as the best fit as it was the most parsimonious model.

The linear covariate for time had a weak negative effect on the probability of obtaining photographs of mangy foxes, meaning that the apparent prevalence of sarcoptic mange slightly decreased over the study period. The interaction between season and distance to settlement had a negative effect in the winter season; and thus, mangy fox events occurred more frequently closer to human settlements during this season (Table 3). The credible intervals of the main effects of distance to settlement and season included zero, and therefore, we were uncertain of the signs of these effects.

**Table 3:** Parameter estimates form the best model (see Table 2) explaining the presence of red foxes with mange-compatible lesions in southeastern Norway.

	Estimate	l-95% CRI	u-95% CRI	Ȓ
Intercept	-3.90	-5.74	-1.86	1
Time	-0.04	-0.11	-0.01	1
Dist_Settle	-0.14	-0.88	0.54	1
SeasonW	0.14	-0.78	1.69	1
Dist_Settle:SeasonW	-0.62	-1.24	-0.03	1
Grid_id(sd(Intercept))	2.14	1.60	2.86	1
Time period(sd(Intercept))	0.77	0.13	2.36	1

l-CRI and u-CRI refer to 95% lower- and upper credible intervals, and Ȓ refer to Gelman and Rubin´s converge diagnosis.

Given the results, we investigated whether the number of red fox events close to human settlements varied during the winter period. We created a binary variable indicating whether a fox event occurred closer than the mean distance to settlement or further from it. We used this variable as a response in a linear regression in a Bayesian framework. We used season as an explanatory variable and grid IK as a random effect. However, we did not find an effect of season on the distribution of red fox events in relation to human settlements (Linear regression estimate: -0.10, CRI [-0.83, -0.63]).

### Spatiotemporal scan analysis

**Fig 4:**
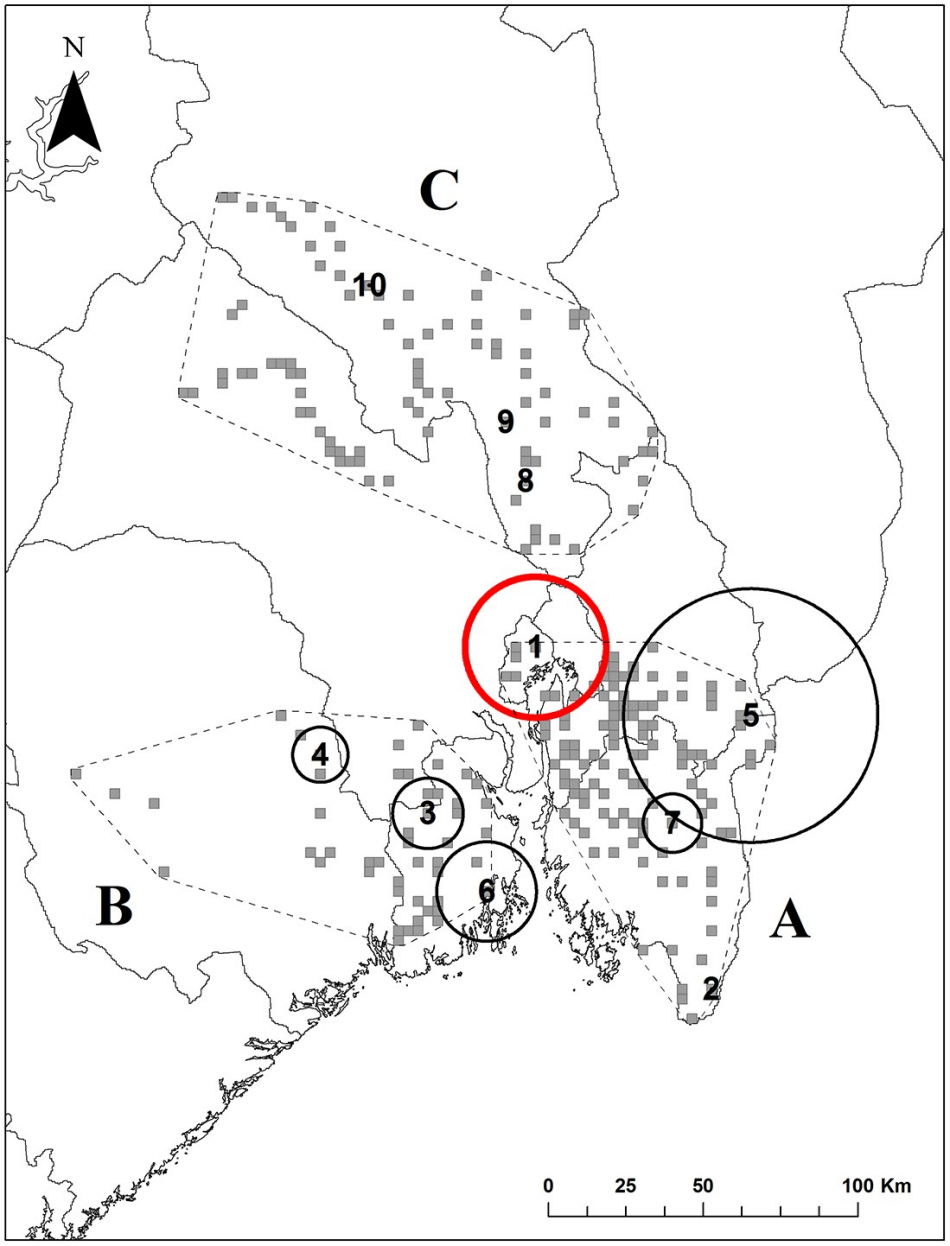
Geographic distribution of significant clusters of red foxes potentially infected with sarcoptic mange identified by scan analysis. The circles define the size of the clusters and the numbers refer to the ID of each cluster (see Table 4 for further details). The red circle represents the cluster with the highest likelihood ratio.

The spatiotemporal scan analysis produced nine significant clusters of potentially infected animals (Table 4, Fig 4). The cluster with the highest likelihood ratio persisted for five time periods (i.e. 30 months) and nine cells with a total radius of 22.71 km. The locations included in this cluster were closer to human settlements than the rest of the locations (Linear regression estimate: -2.84, CRI [-4.9, -0.82]). This cluster presented a difference of likelihood ratio of 177.516 compared with the second most likely cluster. The likelihood ratio decreased again dramatically from cluster four to cluster five. There is a possibility that repeated photographs of the same individual could cause four of the clusters that included only one camera location and only one period of six months. Nevertheless, these four clusters are still important areas to be consider in the future as potential areas of risk, since they could be precursors of bigger outbreaks. However, given the spatial independence of our data and the independence of the time periods, the remaining clusters that had more than one camera location and several periods most probably included several different individuals.

**Table 4:** Spatiotemporal clusters of red foxes potentially infected with sarcoptic mange detected by the scan analyses.

Cluster	Radius (km)	Duration (Months)	Start date	End date	Number Cells	LLR	P_value	Observed Cases	Expected Cases
**1**	**22.71**	**30**	**October_2013**	**March_2015**	**9**	**317.556**	**<0.001**	**391**	**94.41**
2	0	6	October_2014	March_2015	1	177.516	<0.001	71	3.31
3	11.36	12	October_2012	September_2013	8	160.737	<0.001	124	16.20
4	8.90	24	October_2012	September_2014	3	145.155	<0.001	182	41.37
5	40.94	6	October_2011	March_2012	40	58.246	<0.001	65	12.70
6	16.05	6	April_2012	September_2012	3	28.692	<0.001	8	0.22
7	9.44	12	October_2012	September_2013	3	28.595	<0.001	11	0.47
8	0	3	April_2015	June_2015	1	25.102	<0.001	7	0.19
9	0	6	October_2013	March_2014	1	24.675	<0.001	14	1.14
10	0	6	April_2014	September_2014	1	6.849	0.437	5	0.58

Radius of the circular cluster, duration, starting and ending date, number of cells included in the cluster, likelihood ratio values and p-values. “Observed cases” are the actual numbers of photographed events of foxes with mange-compatible lesions in that cluster, while the “Expected cases” are numbers expected conditioning on the total number of cases (see Kulldorff et al. 2005 for a detailed description)

## Discussion

Our study showed that red foxes with mange-compatible lesions were more frequently located closer to human settlements during winter when compare to healthy red foxes. Human settlements are a source of easily available food for several wildlife species, including the red fox [37, 60, 61]. In some cases, this source of food can fulfil the daily requirements of one pair of foxes [28]. Anthropogenic food is most important during the winter period, when prey availability is low [62–65], and it may be particularly important for foxes affected by sarcoptic mange. Several studies have shown that sarcoptic mange deteriorates the body condition of the host [5, 66, 67]. Accordingly, a poor body condition may affect the ability of the red fox to hunt live prey or locate and fight for carrion, and therefore induce foraging close to humans (see [68] for an example with coyotes). Balestrieri et al. [67] suggested that a source of bias in their study was the tendency of mangy foxes often found close to villages looking for easy food. The results of our logistic models are in accordance with our spatiotemporal scan analysis results; the cluster with the highest likelihood encompassed cells closer to human settlements than the rest of the cells within the study areas (-2.84 kilometers, CRI [-4.9, -0.82]). However, it is also important to consider alternative, but not mutually exclusive, mechanisms, such as the possibility that heavy use of anthropogenic food sources that tend to occur in concentrated points in areas close to settlements may facilitate scabies transfer between foxes. We did not detect a variation in the number of red fox events closer to human settlements during the winter; therefore, we discarded a density dependent transmission of the disease. This dissociation between the population density and the transmission of sarcoptic mange have been demonstrated in other places [26, 69]. In the other hand, concentrated anthropogenic food can facilitate an increase in the contact rate independent of density, for example by increasing overlapping among home ranges especially during winter. This pattern is a frequency dependant transmission of the disease (see Begon et al. [70] for a thorough definition of the terms). This transmission mechanism has previously been shown in other urban fox population [69]. The large scale data used in this study does not permit a finer scale analysis of the actual mechanisms, but it clearly identifies important areas for future investigations.

The spatiotemporal scan analyses detected nine significant clusters of mange-compatible lesion observations, among which six included three or more grid cells. The clustered patterns suggest that the dynamics of sarcoptic mange in this red fox population is characterised by small-localized outbreaks that emerge and disappear rather than sporadic events in the population. This pattern of local outbreaks has been observed in other populations where mange is enzootic, like in Britain [24] or Spain [26]. Furthermore, the apparent prevalence of the disease in our study fluctuated over time below ca 11% (Fig 3) with peaks that corresponded in time to some of the potential clusters, including the one with the highest likelihood. Other studies have found similar patterns of mange prevalence in other species like chamois or Iberian ibex [3, 71]. Currently, 30 years after the epizootic outbreak of sarcoptic mange in Fennoscandia, the multiple clusters and fluctuations in the disease apparent prevalence suggest that the disease is in an enzootic state with low prevalence (average 3.16 ± 0.56%). However, this prevalence might be somewhat underestimated in our study, as camera traps only allow for the detection of visually evident symptoms (e.g. alopecia). Hence, we failed to identify infected foxes in early stages of the disease with non-symptomatic infections. The degree of infection can reveal important insight on the dynamic of the disease [7]. However, photographic material is not always reliable when assessing the stage of the infection of an individual. We did not include a degree of infection in our analyses but since we only used clear cases of mange-compatible lesions, we can assume that these individuals were in advanced stages of the disease. We believe that our data accurately reflect spatiotemporal patterns of clinically advanced cases of sarcoptic mange. Regarding the relationship with human settlements, the detriment in body condition occurs in the last stages of the disease [17]; that is the moment when the animal may be more dependent on easily available food. Furthermore, analysing the spatiotemporal variation only in severe cases still allows for detecting potential outbreaks given that the relative proportions of individuals in early and later stages of the disease is relatively constant.

It is important to highlight the potential weaknesses of the use of camera traps for disease epidemiology assessment. Given the nature of the data, we were not able to include variables like sex or age of the photographed individuals. Previous studies showed age specific prevalence of sarcoptic mange in red foxes [69] and sex specific infection rates in coyotes [7]. We did not identify any pup with mange-compatible lesions, but the restricted movement of young individuals [69] might decrease their detectability by camera traps.

The actual origin of the lesions in the animal represent another limitation in the use of camera traps as other causes of hair loss might be confused with sarcoptic mange. Fungal infections known as dermatophytosis can cause alopecia in red foxes [72, 73] but this is not common and has never been reported in Norway. Notoedric mange caused by the mite *Notoedres cati* has symptoms similar to sarcoptic mange, and it affects mainly felines [1]. Other mites like *Demodex canis* affect dogs and has not been reported in foxes [74]. Hence, even though the mange-compatible lesions reported in our study (e.g. Fig 2) are probably caused by the mite *Sarcoptes scabiei*, we recommend that camera trap surveys are accompanied by serological tests of samples like ELISA test [75].

Field surveillance of wildlife diseases is imperative for the management of potential threats both to wildlife and human well-being [76, 77]. The occurrence of mangy foxes in the vicinity of human settlements and the apparent clustered pattern of the disease raise important implications for the management of the Scandinavian red fox population. Clusters appearing close to human settlements might represent a source of contagiousness for domestic animals, livestock or other wildlife [43]. Tools like the StatScan software allow for prospective analyses of surveillance data in order to detect potentially emerging clusters.

Here we show that camera traps are an effective tool to use in surveillance of wildlife diseases with visually apparent symptoms like sarcoptic mange. Oleaga et al. [5] also illustrated the usefulness of camera traps for detecting mange in wolves and red foxes, and other methods such as thermal imaging cameras are on the rise [66, 78]. Nonetheless, surveillance in the field is only a first step towards a holistic monitoring of a wildlife disease, and serological and genetic analyses of the pathogen should accompany them [77, 79]. These analyses can reveal the directionality of the disease transfer (e.g. prey to predator, intraguild contagion) [79], and can increase the accuracy of prevalence estimations.

## Supporting information

S1 Appendix Dataset. Red fox events detected by camera traps from 2010 until 2015 in the southeast of Norway and covariates used in the study.
(XLSX)
